# Shifts in the Lung Microbiota and Antibiotic Resistance Genes Occur With Aging in Patients With Lower Respiratory Tract Infections

**DOI:** 10.1155/bmri/9038281

**Published:** 2026-04-06

**Authors:** Xiaoming Chen, Tingyan Dong, Dongliang Wu, Quanguan Pan, Jinhua Wei, Guangwen Liang, Zetai Lin

**Affiliations:** ^1^ Department of Pediatrics, Huangpu People′s Hospital of Zhongshan, Zhongshan, Guangdong, China; ^2^ Research Center for Healthy Aging and Social Economic Development, Guangzhou College of Commerce, Guangzhou, Guangdong, China; ^3^ Department of Pulmonary and Critical Care Medicine, Huangpu People′s Hospital of Zhongshan, Zhongshan, Guangdong, China

**Keywords:** antibiotic resistance genes (ARGs), correlation analysis, lower respiratory tract infections (LRTIs), lung microbiota, targeted next-generation sequencing (tNGS)

## Abstract

Lower respiratory tract infections (LRTIs) are a leading cause of critical illness and mortality. The lung microbiome represents an important reservoir for the exchange of antibiotic resistance genes (ARGs). The pathogenic microbes remain poorly understood among different age groups, including children (0–17 years), youth (18–39 years), middle‐aged adults (40–64 years), and older adults (65–99 years). We conducted a retrospective study of 699 bronchoalveolar lavage fluid (BALF) samples from LRTI patients aged 30 days to 99 years. The differences in the lung microbiome and ARG expression shift with age were evaluated based on targeted next‐generation sequencing (tNGS) results. Correlation analysis revealed that age had a strongly positive correlation effect on the relative abundances of *Candida albicans*, *Candida glabrata*, *Corynebacterium striatum*, and *Stenotrophomonas maltophilia*. Meanwhile, age had a largely negative correlation effect on *Enterococcus faecium* and *Mycoplasma pneumoniae*. We found that ARG expression was significantly higher in adults compared with children. The beta‐lactam ARG TEM was the most abundant, and the primary carrier of ARGs was *Streptococcus* in the LRTI microbiota. The proportion of adults expressing beta‐lactams, aminoglycosides, and phenicol antibiotic types was higher compared to children. Our results indicated that ARGs in the human LRTI microbiota accumulate and become more complex with age, as older groups tend to harbor the highest abundance of these genes. Collectively, these results presented the respiratory tract core microbiota and ARGs in different age groups, supplying a foundation for microbiome‐targeted interventions and emphasizing the potential of tNGS to improve clinical diagnosis.

## 1. Introduction

Lower respiratory tract infections (LRTIs) rank as a major cause of morbidity and mortality across the globe [1]. Their impact is particularly significant among children and the elderly. Multiple studies have explored the causes of LRTI in children and the elderly [2], yet only a handful of them have specifically examined the age‐related differences between microbiome features and respiratory symptoms. Assessing the microbiological origins of LRTI in this demographic is difficult when relying on conventional microbiological diagnostic methods [3]. Moreover, samples from the upper airways may not accurately represent the disease state in the lungs. Targeted next‐generation sequencing (tNGS) for pathogen surveillance presents a chance to surmount the limitations of culture‐based methods. Different from traditional infectious disease diagnostic techniques, tNGS can conduct a broad screening for various potential pathogens, such as bacteria, viruses, and fungi, all within a single test [4]. It allows for a comprehensive evaluation of the pathogen landscape in the lower airways and provides valuable epidemiological data [5].

Previous studies on the gut microbiome have indicated a link between age and the composition, along with the burden of antibiotic resistance genes (ARGs). Cumulative exposures might affect the resistance characteristics of endogenous microbial communities [6, 7]. When subjected to antibiotics, the microbiota not only reacts via its own resistance mechanisms but also enhances and disseminates ARG through transformation, transfer, and recombination, ultimately forming a colony with an antibiotic‐resistant phenotype [8]. This situation poses a substantial risk of increased antibiotic resistance in human pathogens, which has become a serious global public health concern as it renders previously reliable antibiotics ineffective [7, 9]. Similar to human gut microbiota, the human lung microbiota is an ecosystem with a complex and dynamic balance and could be influenced by antibiotics [10]. Despite these findings, only a limited number of studies have both described the microbial ecology of LRTI and detected the age‐related expression of ARGs [11, 12]. In this study, we also investigate the relationship between advanced age and a higher prevalence of ARGs in the lung microbiome.

By culture‐independent tNGS diagnostics, we investigated the prevalence and age distribution of lower respiratory pathogens in a single‐center cohort of patients with LRTI. Considering that the respiratory microbiome experiences significant alterations in early childhood [13] and is increasingly acknowledged to have a crucial role in the pathogenesis of respiratory infections [14], we postulated that age‐related changes in the respiratory microbiome could account for the differences in different age groups. In this research, we utilized tNGS to profile the lung microbiomes of LRTI patients in different age groups, including children, adolescents, young adults, and the elderly, to identify microbiome characteristics associated with age variation. Our objective is to delve into findings that can deepen our comprehension of the lung microbiome and resistome in patients with LRTI across their lifespan. This exploration may have significant implications for clinical management.

## 2. Materials and Methods

### 2.1. Patients and Sample Collection

We conducted a retrospective review of LRTI patients admitted to Huangpu People′s Hospital of Zhongshan between March 2024 and March 2025. The attending physicians determined whether a patient had LRTI according to clinical manifestations and imaging examination results. The inclusion criteria were as follows [13]: patients presenting with distinct symptoms of LRTI, specifically the following: (1) For pneumonia patients, any of the following symptoms or signs should be present: fever (higher than 38°C), breathing suspension, shortness of breath, bradycardia, wheezing, coughing, snoring, and chest imaging revealing new or progressive exudation, solid shadows, cavities, or pleural effusions. (2) For tracheitis or tracheobronchitis patients, two of the following symptoms or signs needed to be observed: cough with increased sputum, hoarse voice, wheezing, respiratory distress, apnea, or bradycardia, while the patient showed no clinical symptoms or X‐ray evidence of pneumonia. (3) Other LRTIs identified through lung radiographic examination, such as lung abscess or empyema. (4) Patients who consented to undergo the tNGS examination. The exclusion criteria were as follows: (1) patients who declined to undergo the tNGS examination, (2) samples that failed to meet the quality control requirements during the tNGS detection process, and (3) patients with incomplete clinical and laboratory data.

Based on our inclusion and exclusion criteria, a total of 699 patients with bronchoalveolar lavage fluid (BALF) were selected for further in‐depth and detailed assays following a composite reference standard (the final clinical diagnosis). This standard incorporated clinical signs and symptoms, microbiological evidence, imaging results, and clinical adjudication. These patients were recruited from the inpatient area of Zhongshan Hospital in China. In this study, a total of 699 samples were analyzed for tNGS sequence. Among them, 44 samples were from children(aged 0–17 years), 31 samples were from youth (aged 18–39 years), 237 samples were from middle‐aged patients (aged 40–64 years), and 387 samples were from older patients (aged 65–99 years).

### 2.2. Extraction of Nucleic Acid and Preparation of Library

According to the instructions of the DNA Miniprep Kit from the manufacturer (ZymoBIOMICS, Zymo, R2002), 5 mL of BALF was separately collected for DNA extraction. The extracted nucleic acids were quantified and then amplified via polymerase chain reaction (PCR). The multiplex PCR–based targeted gene sequencing technique was employed to identify the targeted pathogens. The PCR assay incorporated a set of multiple primers along with primers regulated by an internal process. The PCR reaction was carried out in the following manner: initial denaturation at 95°C for 3 min; followed by 25 cycles, each cycle including denaturation at 95°C for 20 s, annealing for 20 s, and extension at 60°C for 4.5 min; and a final extension at 72°C for 5 min. The amplified product was processed using the magnetic bead method for amplification and purification, and targeted capture and sequencing libraries were constructed. The qualified DNA libraries were then sequenced on the Illumina high‐throughput sequencing platform. The sequencing data were automatically normalized based on the amplification calibration coefficient. Reads shorter than 60 bp or those with nonspecific primer binding were removed through filtering, and the clean reads were utilized for sequencing analysis.

### 2.3. tNGS

A total of 199 pathogens (Table S1) associated with respiratory tract infections were incorporated into the identification model for diagnosis. Initially, the DNA sequences were chosen as the targeted fragments. Multiplex PCR was employed to amplify and enrich the targeted gene sequences. A sequencing library was created by attaching sequencing connectors to the purified PCR products and purifying them using DNA purification magnetic beads. Targeted gene sequencing (300 cycles) was carried out on an MGI200 system in the FGS‐SE60 test mode. The MiSeq Reporter software generated Fast‐q files. The offline data were identified and counted, and reads with a double‐end length greater than 60 bp were retained. Among the high‐quality data, reads with a length of less than 60 bp at either end, single‐end primer recognition, or nonspecific primer binding were rechecked and removed. Clean read pairs were obtained for identification and sequence alignment. Prior to aligning with pathogen reference sequences, the clean read pairs were first aligned to the human reference genome. Read pairs that aligned with the human genome were discarded to minimize host DNA contamination. The remaining read pairs were then aligned to pathogen reference sequences. Read paired counts for each pathogen were generated for further analysis.

### 2.4. Analysis of ARGs

The clean sequencing data were aligned with the human host reference genome by means of the STAR software, and the host reads were then eliminated. For all nonhuman source reads, the UBLAST strategies (with an *E* − value ≤ 10^−7^) were employed to search against the well‐organized structured ARG database SARG. This SARG database incorporated ARDB, CARD, and the most recent NCBI‐NR database. The criteria for ARG identification were an alignment length of at least 75 nt and an alignment identity rate of 80%. To conduct a more in‐depth comparison of the ARG distribution in different BALF specimens, we standardized the abundance of ARG. We expressed it as the number of ARG copies per cell, following the formula described in a prior study. The heat map for ARGs was generated using self‐developed R scripts. The calculation of “ARGs proportion changing with age” is the number of samples present at each age divided by the total number of samples at that age, which is the detection frequency.

### 2.5. Statistical Analysis

The correlation analysis was conducted by performing a Spearman correlation analysis between the log10 (abundance) of pathogens and the age values. Statistical significance was determined when the *p* value was less than 0.05, with two‐tailed hypothesis tests employed. For the comparison between two groups, the Wilcoxon rank‐sum test was used, except for binary variables, for which Fisher′s exact test was applied. All statistical analyses were carried out using the R software Version 4.1.2.

## 3. Results

### 3.1. Study Populations

A total of 699 patients (481 males, 31.2%; 218 females, 68.8%) with LRTI were included in this study. The mean age was 67.7 years. These subjects were divided into four groups: 0–17Y (44 children, 0–17 years old), 18–39Y (31 young patients, 18–39 years old), 40–64Y (237 middle‐aged patients, 40–64 years old), and 65–99Y (387 older patients, 65–99 years old). Ninety‐two percent of the patients received antibiotics prior to sample collection. All patients underwent bronchoscopy to collect BALF within 48 h for tNGS analysis, and the pathogen detection results are shown in Table S2.

### 3.2. Discrepancies of LRTI Microbiome in Different Age Groups

To investigate differences in the age‐related pathogens, we determined the distribution of the Top 20 most frequently detected pathogens across the different age groups. The frequently encountered bacterial pathogen in children (0–17Y) was *Mycoplasma pneumoniae* (33/44, 75%), followed by *Staphylococcus aureus* (6/44, 13.6%) and *Streptococcus pneumoniae* (6/44, 13.6%). The most common viruses in children (0–17Y) were Human Herpesvirus 7 (5/44, 11.4%) (Figure 1a). The most common bacterial pathogen in young patients (18–39Y) was *Corynebacterium striatum* (16/31, 51.6%), followed by *Streptococcus intermedius* (9/31, 29.0%). The most common viruses were Human Herpesvirus 7 (10/31, 32.3%), followed by Human Herpesvirus 1 (7/31, 21.6%). *Candida albicans* (7/31, 22.6%) was the most common fungus (Figure 1b). The Top 3 common bacterial pathogens in middle‐aged patients (40–64Y) were *Corynebacterium striatum* (113/237, 47.7%), followed by *Pseudomonas aeruginosa* (60/237, 25.3%). The most common viruses were Human Herpesvirus 7 (94/237, 39.7%), followed by Human Herpesvirus 5 (68/237, 28.7%). The most common fungi were *Candida albicans* (31/237, 13.1%) (Figure 1c). The two common bacterial pathogens in older patients (65–99Y) were *Corynebacterium striatum* (207/387, 53.5%), followed by *Klebsiella pneumoniae* (107/387, 27.6%). The most common viruses were Human Herpesvirus 1 (120/387, 31.0%). The most common fungus was *Candida albicans* (109/387, 28.2%) (Figure 1d).

Figure 1Distribution of pathogens in LRTI patients (a) aged 0–17 years (0–17Y), (b) aged 18–39 years (18–39Y), (c) aged 40–64 years (40–64Y), and (d) aged 65–99 years (65–99Y). (e) Comparison of the abundance of pathogens in each group. The level of significance for the Wilcoxon rank‐sum test is  ^∗^ *p* < 0.05 and  ^∗∗^ *p* < 0.01. (f) Bar chart of the Top 20 common species in the different age groups. Each color bar represents one species.

**Figure:**
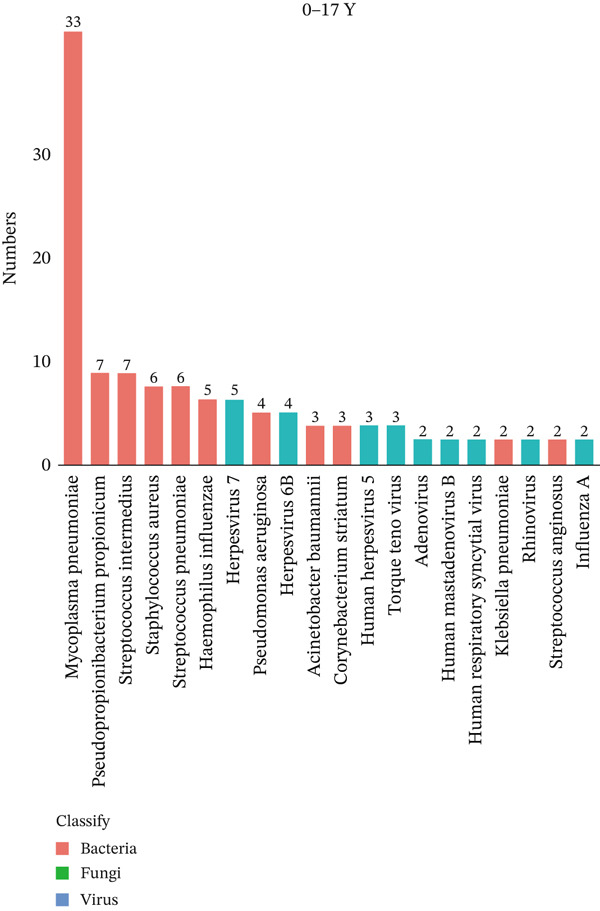
(a)

**Figure:**
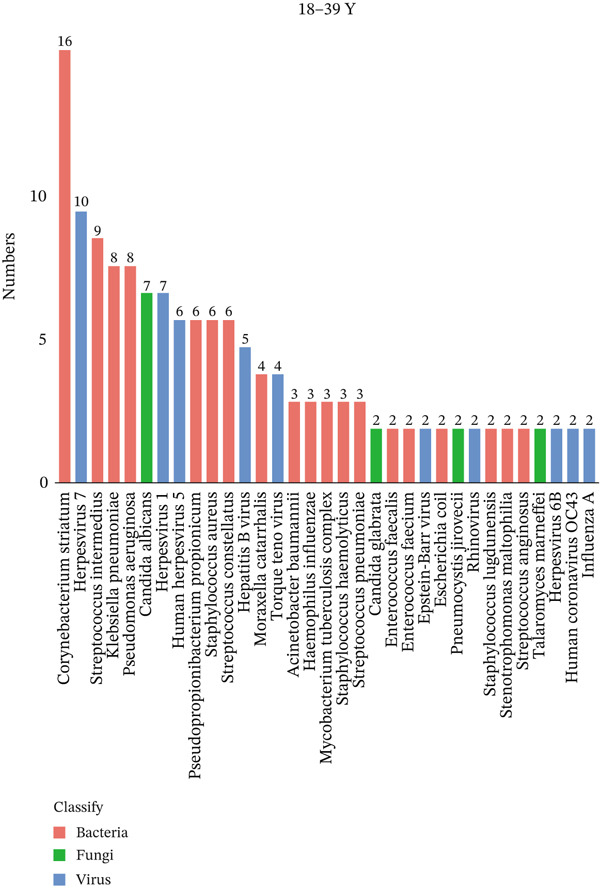
(b)

**Figure:**
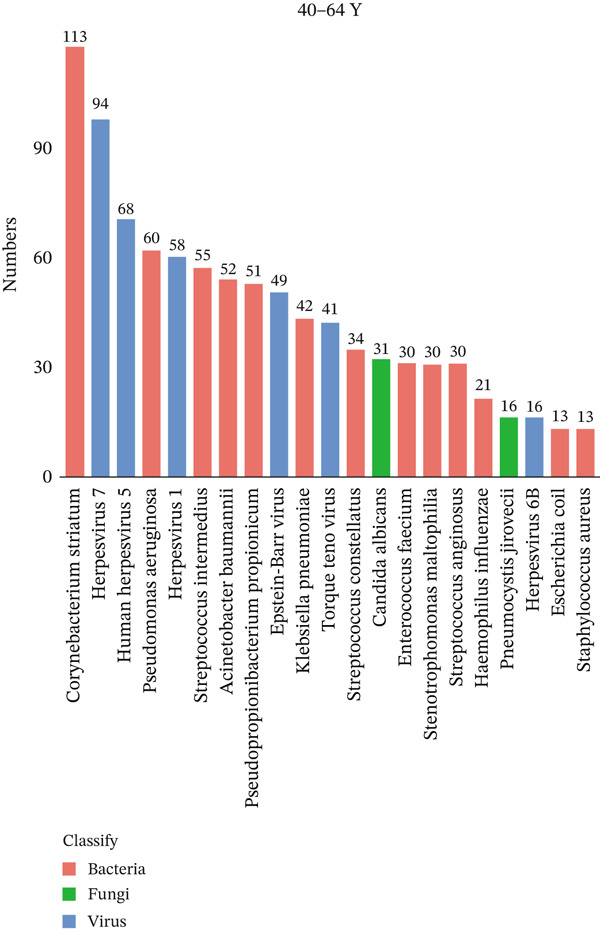
(c)

**Figure:**
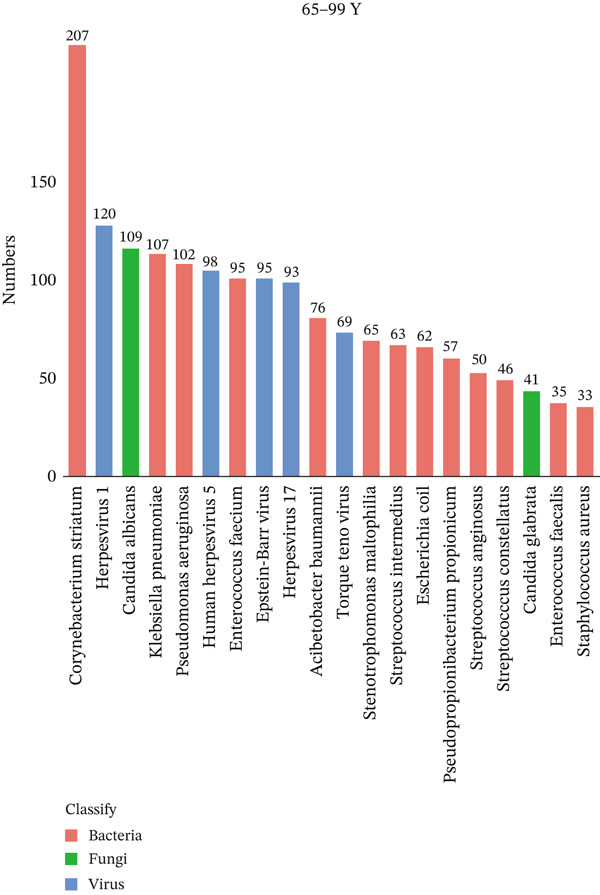
(d)

**Figure:**
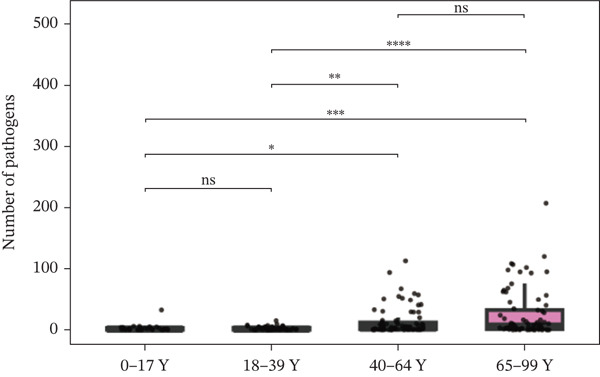
(e)

**Figure:**
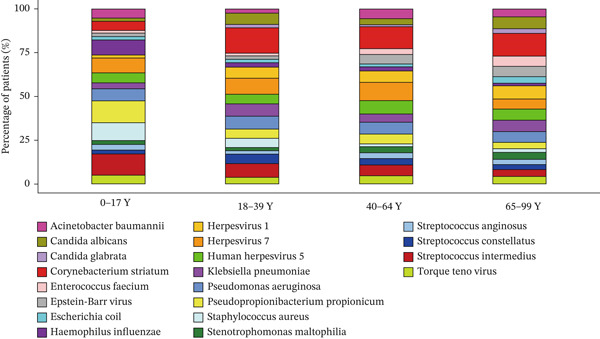
(f)

To compare the microbiome abundances of different age groups, we calculated the number of pathogens in each group based on sequencing coverage. The number of pathogens in the children group (0–17Y) and the young group (18–39Y) was significantly lower (*p* < 0.05) than that in the adult groups (40–64Y and 65–99Y; Figure 1e). We analyzed the Top 20 common pathogens in these four groups in the hot program (Figure 1f). The most commonly detected pathogens were *Corynebacterium striatum*, Human Herpesvirus *7*, *Acinetobacter baumannii*, and *Streptococcus anginosus*. These 20 pathogens occurred in all ages, which contributed to the LRTI progression.

### 3.3. Typical Resistance Gene Types in Various Age Groups

The Top 20 most abundant ARG types varied among the different age groups (Figure 2), with the exception of TEM, which was the top abundant type in all groups (Figure 2a). The mechanism of resistance gene type TEM is beta‐lactam and tetracycline antibiotics, and TEM belongs to the gene family of tetracycline‐resistant ribosomal protection proteins. ermB was the second most abundant gene type. It was macrolide–lincosamide–streptogramin (MLS) B resistance genes that alter the antibiotic target and belong to the gene family of erm 23S ribosomal RNA methyltransferases (Table S3). In the different age groups, different resistance gene types were observed. 23srRNA2063A>G, TEM, and ermB ARGs have the highest abundance in the children group (0–17Y). Meanwhile, TEM, ermB, and tetM in the 18–39‐year‐old group were the most abundant resistance gene types, while TEM, AAC, and ADE were found to have the highest abundance in the 40–64‐ and 65–99‐year‐old groups. Other categories of drugs and resistance mechanisms are listed in Table S3.

Figure 2(a) The cumulative bar graph represents the distribution of the Top 20 antibiotic resistance types in the different age groups. (b) Comparison of the abundance of ARGs in each group. (c) Significantly difference of ARGs and virulence factors in four age groups. (d) Comparison of the distribution of the relative abundance of antibiotic types in the different age groups. (e) The cumulative bar graph represents the distribution of the Top 20 virulence factors in the different age groups. (f) Comparison of the abundance of virulence factors in each group. The level of significance for the Wilcoxon rank‐sum test is  ^∗^ *p* < 0.05,  ^∗∗^ *p* < 0.01, and  ^∗∗∗^ *p* < 0.001.

**Figure:**
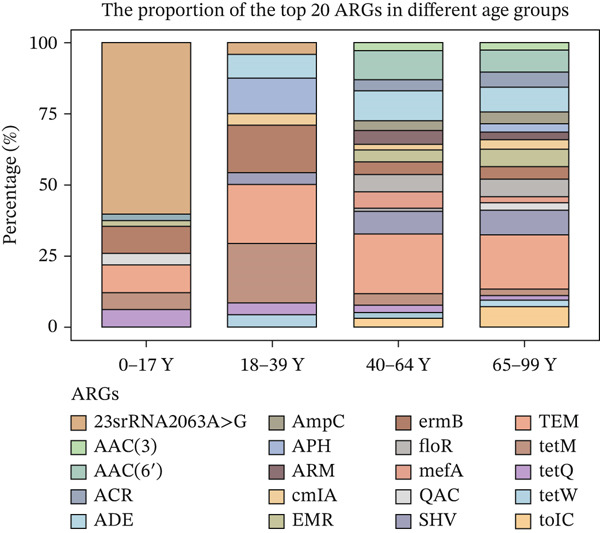
(a)

**Figure:**
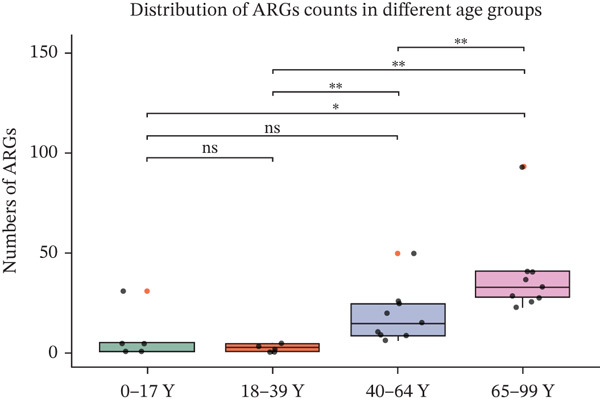
(b)

**Figure:**
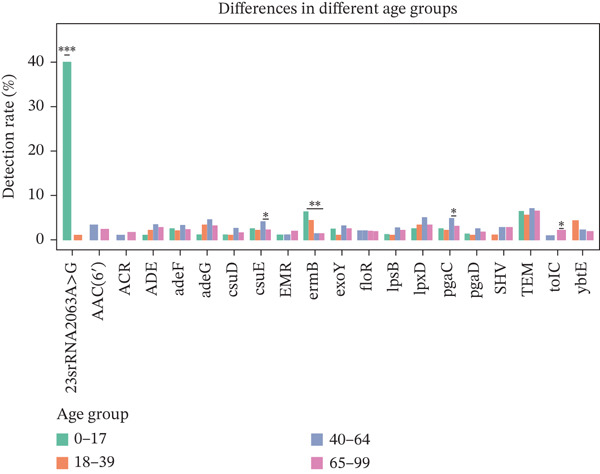
(c)

**Figure:**
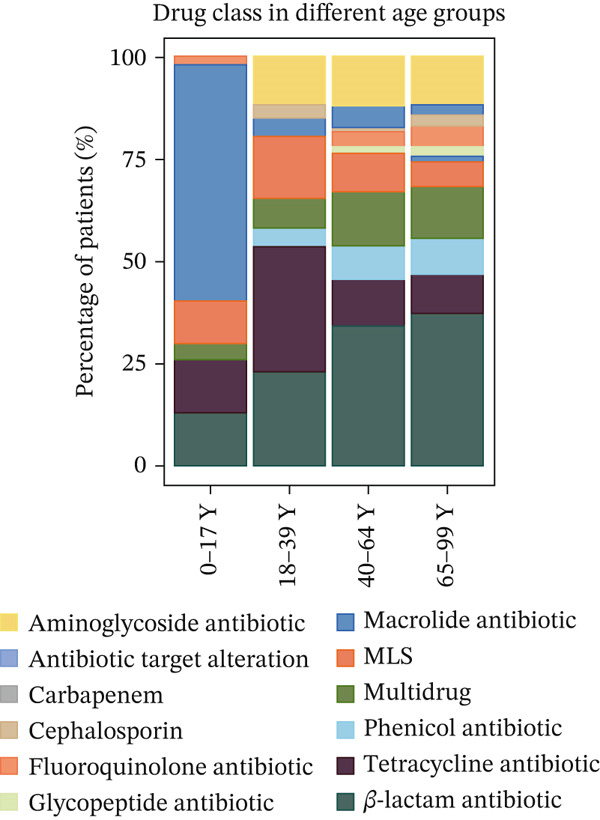
(d)

**Figure:**
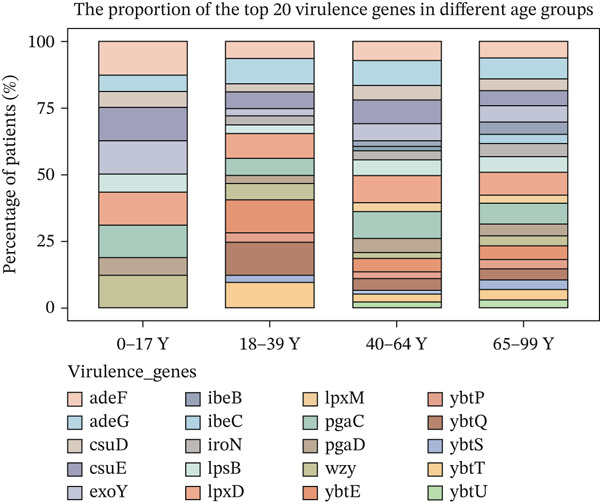
(e)

**Figure:**
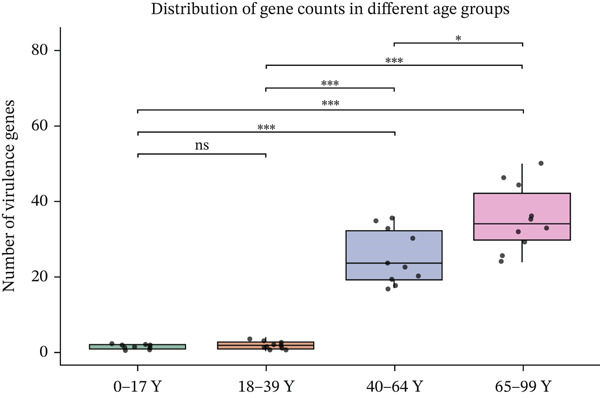
(f)

To compare the ARG abundances of different age groups, we calculated the number of ARGs in each group based on sequencing coverage. The number of ARGs in the children group (0–17Y) was significantly lower (*p* < 0.05) than that in the older group (65–99Y). There was a significant difference (*p* < 0.05) between the adult groups, and the ARG abundances increased with age (Figure 2b). Our results indicate that ARGs in the human lung microbiota accumulate and become more complex with age, with older groups harboring the highest abundance of these genes. 23srRNA2063A>G in the children group (0–17Y) and ermB and tolC in the adult group were significantly abundant (Figure 2c). We mapped each resistance gene type to its corresponding antibiotic and calculated the sum of the relative abundances of antibiotic types (Figure 2d). We observed that abundant antibiotic resistance types in lung bacterium were beta‐lactam antibiotic, followed by MLS and tetracycline resistance in all the age groups. The trend of beta‐lactam antibiotic consumption gradually increased with age. Aminoglycosides and multidrug antibiotics were observed to be significantly more abundant in the adults (aged 18–99 years) than in the children (aged 0–17 years). In contrast, the macrolide antibiotic (23srRNA2063A>G, most abundant), due to the high abundance of *Mycoplasma pneumoniae*, was markedly more abundant in children than in adults. Abbreviations of antibiotic types are listed in Table S3.

We also analyzed the most significantly abundant virulence factors in each age group. adeG, csuE, lpxD, and pgaC were the most common and abundant types in different age groups (Figure 2c). csuE and pgaC were significantly different in the 40–64Y and 65–99Y groups (Figure 2e). Similar to the ARGs, the number of virulence factors in the children group (0–17Y) was significantly lower than that in the adult groups (40–64Y and 65–99Y; Figure 2f). The number of virulence factors gradually increased with age.

### 3.4. Analysis of LRTI Microbiota and ARG Shift With Age

To determine whether certain pathogens were associated with age, we compared the number of cases of each pathogen in patients with LRTI. The prevalence of 16 species was statistically different (*p* < 0.05) across age groups in patients with LRTI: *Alphapapillomavirus* 16, *Chlamydia trachomatis* (*p* < 0.01), *Corynebacterium striatum* (*p* < 0.001), *Cryptococcus*, *Cryptococcus neoformans*, Hepatitis B virus, Human parvovirus B19, human coronavirus OC43, human coronavirus NL63, *Mycoplasma pneumoniae* (*p* < 0.001), *Mycobacteroides abscessus*, *Streptococcus intermedius*, *Streptococcus dysgalactiae*, *Streptococcus constellatus*, and *Talaromyces marneffei* (Figure 3a).

Figure 3Correlation between age and the relative abundance of pathogens in different age groups. (a) Pathogens were significantly different in different age groups. (b) The observed age‐related pathogen differences could be verified due to the continuity of the sampling age distribution. (c) The trend of the proportion of ARGs changing with age.

**Figure:**
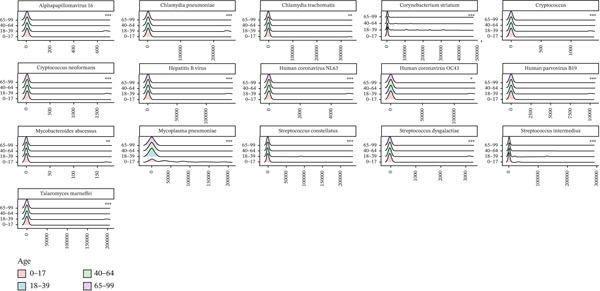
(a)

**Figure:**
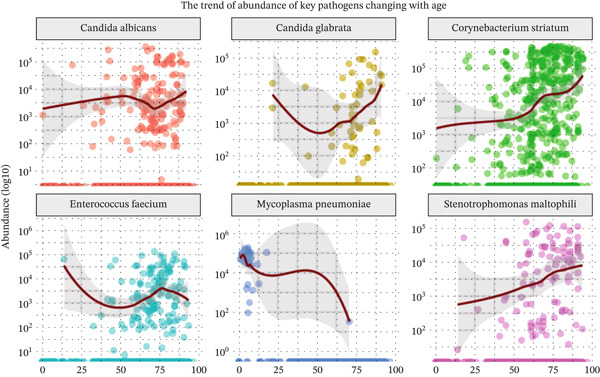
(b)

**Figure:**
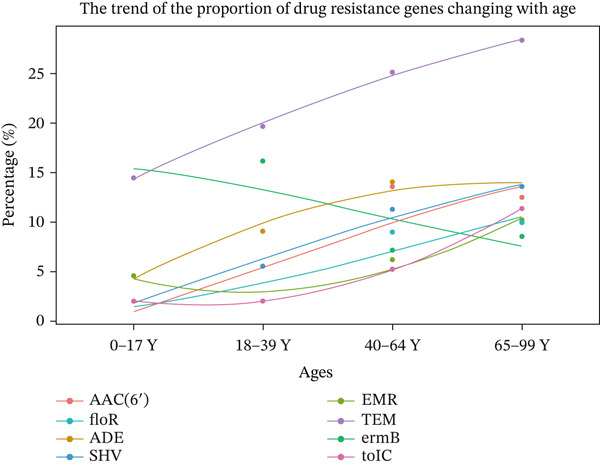
(c)

By using the Spearman correlation analysis between the log10 (abundance) of pathogens and the age values, we found a strongly positive correlation effect of age on the relative abundances of *Candida albicans*, *Candida glabrata*, *Corynebacterium striatum*, and *Stenotrophomonas maltophilia*. Meanwhile, age had a largely negative correlation effect on *Enterococcus faecium* and *Mycoplasma pneumoniae* (Figure 3b). The most frequently detected pathogens in children (0–17Y) were *Mycoplasma pneumoniae*, and the prevalence of these pathogens markedly decreased with increasing age across the adult groups (aged 18–99 years). The fungal pathogens, *Candida albicans* and *Candida glabrata*, markedly increased in patients over 65 years old. Compared with LRTI patients over 65 years old, young patients had lower fungal loads but higher viral loads.

The most frequently detected resistance genes, including ADE, EMR, ermB, SHV, TEM, tolC, and ACE, increased with age (Figure 3c). Our results indicate that ARGs in the human lung microbiota accumulate and become more complex with age, with older groups harboring the highest abundance of these genes.

### 3.5. Representative Antibiotic Resistance Types of Different Bacterial Genera

**Figure 4:**
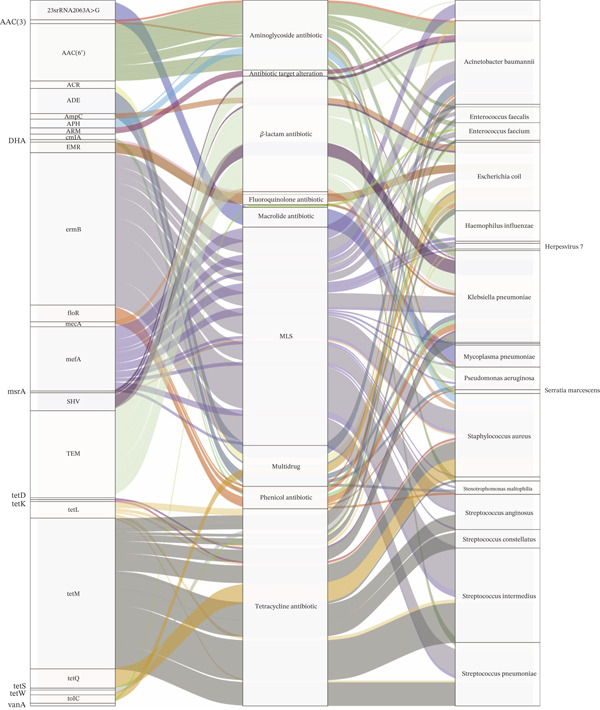
The Sankey diagram shows the connectivity between the most prevalent ARGs and their corresponding species, as well as the drug class. Different colors refer to different species and different drug resistance classes.

To find out which bacterial genera contributed to the ARG reservoir, we performed an association analysis of ARG and bacterial genera and characterized the antibiotic resistance types present in these bacteria. It was found that many species harbored multiple ARGs, and most ARGs were shared among different species (Figure 4). We found that the most predominant genus in the lung microbiota was *Streptococcus*. The Top 5 contributing bacteria were *Streptococcus intermedius*, *Streptococcus pneumoniae*, *Klebsiella pneumoniae*, *Escherichia coli*, and *Acinetobacter baumannii*. Similarly, the main carrier of ARG was *Streptococcus*.

## 4. Discussion

Based on the tNGS results, we were able to identify age‐related pathogens. In patients with LRTI, the overall species abundance of the lung microbiome and ARGs showed an upward trend with increasing age. Specifically, the oldest patients had the highest number of ARGs. These findings enhance our comprehension of the lung microbiome as a potential reservoir of antimicrobial resistance. The lung microbiome, being in close proximity to the respiratory tract and having a direct impact on the local immune environment, may play a crucial part in the development and spread of drug‐resistant respiratory pathogens. Understanding these complex relationships is of utmost importance for developing more effective strategies to combat drug‐resistant respiratory infections and safeguard public health.

In children with LRTI, human herpesvirus in the BALF was the most frequently detected pathogen. This differed from what was previously reported regarding throat swabs and sputum specimens [15]. In the study of pediatric community–acquired pneumonia, *Mycoplasma pneumoniae* was the most common bacterial pathogen detected [16]. We found this microbe in 75% of the children, indicating a relatively high disease burden in most children with LRTI. *Staphylococcus aureus* was detected in 13.6% of the children, suggesting that the significance of incidentally detecting this pathogen in the lower airway may have been underestimated. We noticed age‐related differences in the LRTI microbiome, covering aspects such as species abundance, diversity, and taxonomic composition. Our results are consistent with a previous study showing that the bacterial abundance in the lung microbiome of cystic fibrosis patients rises with age [17]. The species diversity increased in childhood and decreased with older age. This could be associated with our finding of frequent incidental carriage of potentially pathogenic bacteria in the no‐evidence group. The significance of the endogenous respiratory microbiota in both the pathophysiology and diagnosis of critical illness syndromes is being increasingly acknowledged [18]. Our findings indicate that age adjustment should be taken into account in clinical and translational research on the lung microbiome.

In this research, we characterized the reservoir of ARGs in the human lung microbiota at the NGS level. Our findings indicated that these genes were prevalent in the microbiota. Moreover, they were more plentiful and diverse in older individuals compared to younger ones. *Streptococcus* was identified as the main carrier of ARG, among which the ermB genes constituted the most abundant group. Resistance mechanisms were mainly dominated by MLS antibiotics. The variation in ARGs among different age groups might be accounted for by the distinct selection pressures exerted by antibiotics [19]. According to previous studies, there exists a direct relationship between antibiotic usage and the degree of resistance [20]. Antibiotic treatment disrupts the equilibrium between the human host and its various microbes, resulting in the emergence of antibiotic‐resistant strains and associated diseases [21, 22]. Thanks to the rapid progress of high‐throughput technology, it has become possible to sequence resistance genes that could not be sequenced on plasmids previously and to detect numerous ARGs in the human lung microbiota [23, 24]. These results indicate that, within the human lung microbiota, the prevalence of ARGs is an age‐dependent cumulative phenomenon, with distinct gene types being more prevalent in individuals of different age groups. Moving forward, future studies should be aimed at elucidating the crucial roles of hosts, carriers, and vectors within the transmission chain and uncovering the underlying mechanisms facilitating the dissemination of ARGs among humans, the environment, and bacteria.

This study has several limitations. Selection bias during patient enrollment and observer bias during LRTI adjudication may have affected our findings. Since this study was a retrospective analysis, it was not feasible to include a sufficient number of BALF samples from pediatric patients. Prior research has shown that NGS can identify pathogens in patients who have taken antibiotics before sample collection, as microbial nucleic acid remains detectable even when the organisms are no longer alive [25]. However, we did not compare the results with traditional methods. A high level of taxonomic abundance or dominance of the airway microbiome does not inevitably imply pathogenicity [26]. In fact, certain bacteria, such as *Streptococcus pneumoniae* and *Haemophilus influenzae*, can be present either as commensals or as pathogens depending on the context [27]. Moreover, some pathogens may cause disease even when they are not the dominant species in the airway [28].

## 5. Conclusion

We show that age is independently related to the detection of certain pathogens and the expression of ARGs in the lung microbiome among a group of patients with LRTI. Our findings indicate that the detection of ARGs in the lower respiratory tract rises across most of the age ranges. Specifically, the oldest patients have the highest number of ARGs that can be detected and expressed at both the individual gene and class levels. These results enhance our understanding of the lung microbiome as a possible reservoir of antimicrobial resistance and emphasize its potential role in contributing to drug‐resistant respiratory infections.

## Author Contributions

Xiaoming Chen was responsible for data curation; writing the original draft, as well as the review; and conducting editing. Tingyan Dong contributed to methodology, formal analysis, and visualization. Dongliang Wu was involved in methodology, resource provision, and investigation. Jinhua Wei participated in writing the review and editing. Quanguan Pan was engaged in data curation and investigation. Guangwen Liang contributed to data curation. Zetai Lin was in charge of conceptualization, supervision, and validation. Xiaoming Chen and Tingyan Dong contributed equally to this work and share first authorship.

## Funding

No funding was received for this manuscript.

## Disclosure

All authors have read and consented to the published version of the manuscript.

## Ethics Statement

This study involves human participants. The study was approved by the Ethics Committee of Huangpu People′s Hospital of Zhongshan (number: hpyy‐wwlw‐2025‐001). The participants provided written informed consent to participate in this study.

## Conflicts of Interest

The authors declare no conflicts of interest.

## Supporting Information

Additional supporting information can be found online in the Supporting Information section.

## Supporting information


**Supporting Information 1** Table S1: A 199‐pathogen panel for the tNGS.

**Supporting Information 2** Table S2: The pathogens detected in patients with LRTI by the tNGS.

**Supporting Information 3** Table S3: The ARGs detected in patients with LRTI by the tNGS.

## Data Availability

The data that support the findings of this study are openly available in NCBI at http://www.ncbi.nlm/. https://nih.gov/bioproject/PRJNA1304736, reference number PRJNA1304736.
